# Anakoinosis: Communicative Reprogramming of Tumor Systems - for Rescuing from Chemorefractory Neoplasia

**DOI:** 10.1007/s12307-015-0170-1

**Published:** 2015-08-11

**Authors:** Christina Hart, Martin Vogelhuber, Daniel Wolff, Sebastian Klobuch, Lina Ghibelli, Jürgen Foell, Selim Corbacioglu, Klaus Rehe, Guy Haegeman, Simone Thomas, Wolfgang Herr, Albrecht Reichle

**Affiliations:** Department of Internal Medicine III, Haematology & Oncology, University Hospital of Regensburg, Regensburg, Germany; Department of Biology, Universita’ di Roma Tor Vergata, Rome, Italy; Department of Pediatrics, University Hospital of Regensburg, Regensburg, Germany; University Gent, Ghent, Belgium; Department of Internal Medicine III, Hematology and Oncology, University Hospital of Regensburg, Franz-Josef-Strauss-Allee 11, 93053 Regensburg, Germany

**Keywords:** Communication tools, Transcriptional modulation, Metronomic low-dose chemotherapy, Drug repurposing, Tumor heterogeneity, Cellular therapy in situ, Anakoinosis, Acute myelocytic leukemia, Classic Hodgkin lymphoma, Multiple myeloma, Langerhans cell histiocytosis, Renal clear cell carcinoma, Castration-resistant prostate cancer

## Abstract

Disruptive technologies, such as **communicative reprogramming (anakoinosis)** with **cellular therapies in situ** for treating refractory metastatic cancer allow patient care to accelerate along a totally new trajectory and highlight what may well become the next sea change in the care of patients with many types of advanced neoplasia. Cellular therapy in situ consisted of repurposed drugs, pioglitazone plus all-trans retinoic acid or dexamethasone or interferon-alpha (dual transcriptional modulation) combined with metronomic low-dose chemotherapy or low-dose 5-azacytidine, plus/minus classic targeted therapy. The novel therapeutic tools for specifically designing communication processes within tumor diseases focus on redirecting (1) rationalizations of cancer hallmarks (constitution of single cancer hallmarks), (2) modular events, (3) the ‘metabolism’ of evolutionary processes (the sum of therapeutically and intrinsically inducible evolutionary processes) and (4) the holistic communicative context, which determines validity and denotation of tumor promoting communication lines. Published data on cellular therapies in situ (6 histologic tumor types, 144 patients, age 0.9–83 years) in castration-resistant prostate cancer, pretreated renal clear cell carcinoma, chemorefractory acute myelocytic leukemia, multiple myeloma > second-line, chemorefractory Hodgkin lymphoma or multivisceral Langerhans cell histiocytosis, outline the possibility for treating refractory metastatic cancer with the hope that this type of reprogrammed communication will be scalable with minimal toxicity. Accessibility to anakoinosis is a tumor inherent feature, and cellular therapy in situ addresses extrinsic and intrinsic drug resistance, by redirecting convergent organized communication tools, while been supported by quite different pattern of (molecular-)genetic aberrations.

## Introduction

Currently proposed cancer models are favoring the selection of theme-dependent therapeutic targets, i.e., tumor-promoting pathways, specific epitopes etc. [1]. Despite the multifold beneficial effects of classic targeted therapies, the underlying cancer models are stretched to their limits if mixed or poor response occurs at metastatic sites, although the respective targets have been diagnostically proven [2].

One limitation of reductionist cancer models is the theme-dependent attribution of tumor systems objects, i.e., cell compartments, pathologic pathways, oncogenes etc. to distinct tumor features, e.g., hallmarks of cancer, such as inflammation, angiogenesis, immune response etc. [3]. Frequently, genetic heterogeneity, just at metastatic sites, limits validity of reductionist cancer models: Situate validity and denotation of tumor systems participators are systems-and stage-dependently highly diversified [4, 5].

Additionally, changing compositions of the tumor microenvironment during tumor progression and metastatic spread suggest stage-dependent diversification of processes supporting hallmarks of cancer, so called **rationalizations** [6, 7]. The situate diversity and redundancy of such rationalizations leads to the assumption that we cannot precisely predict in developing systems, such as in tumors, the situate validity and denotation of single systems objects, i.e., cells, pathways, and therapeutic targets etc. without administering novel read-out technologies describing their situate functionality [8].

Insufficient treatment response based on reductionist derived tumor models, leads to revising underlying hypotheses. The importance of alternative therapy models may be proven by systematically designing treatment approaches for chemorefractory metastatic tumor diseases. **‘Cellular therapies in situ’** are representing such novel approaches.

Primary aim of cellular therapies in situ is the **communicative reprogramming of tumor systems (anakoinosis)** for diversifying palliative care, moreover, for uncovering novel ways for the induction of tumor cell death. Cellular therapies in situ comprise repurposed drugs [9, 10] with poor monoactivity, but concerted regulatory activity, such as transcriptional modulators (dual transcriptional modulation) [11–13], metronomic low-dose chemotherapy [14], and classic targeted therapies [9].

The theoretical, communication based background has been derived from phase II trials on cellular therapies in situ, summarized in the **‘principles of modular tumor therapy’** [15, 16]. Cellular therapies in situ have the capacity for rededicating functions and identities of cellular compartments or tumor-promoting pathways, thereby attenuating tumor growth (**anakoinosis)** without directly knocking down tumor-promoting pathways or single cell compartments.

The summary on studies using cellular therapies in situ impressively shows, to our knowledge for the first time, that **anakoinosis** reproducibly mediates diversified novel systems behaviors in chemorefractory tumors, and that communicative reprogramming is able to depict and categorize targetable communication tools in refractory tumor systems.

## Material and Methods

**For presenting tumor-associated communication tools as valid therapeutic targets** in systemically pretreated, refractory metastatic cancers or hematologic malignancies, we summarized published clinical trials or compassionate use approaches, which uniquely introduced cellular therapies in situ.

The single components of cellular therapies in situ are showing concerted regulatory activity, why toxicity can be saved [16]. Cellular therapies in situ, presented here, consisted of two major components, **dual transcriptional modulation** [11–13] combined with drugs, which may enhance the activity profile of transcriptional modulators, i.e., metronomic low-dose chemotherapy (angiostatic, immune modulatory) [14] or low-dose 5-azacytidine (epigenetic reprogramming). A third component was added in some trials, namely classic targeted therapies (imatinib, COX-2 inhibitors, lenalidomide and mTor-inhibitors), again in a drug repurposing manner to intensify the angiostatic, anti-inflammatory, and immune modulatory activity profile (Table 1) [9, 10].

**Table 1:** Anakoinosis: designing evolutionary processes in tumor tissues and exploiting and operationalizing their scope with cellular therapies in situ

	Cellular therapy in situ Anakoinosis: Designing evolutionary processes in tumor tissues and exploiting and operationalizing their scope		
Drug repurposing for communication design	Anakoinosis promoting therapeutic tools: ‘Top down’ strategies		
	Dual transcriptional modulation	Metronomic low-dose chemotherapy	Classic targeted therapy
Communication derived tools for evolving tumor systems	Evolving tumor systems • Targets are ubiquitously distributed among tumor, stroma and organ cells; • Stimulating, not blocking	Modulation of • Angiogenesis, immune response, inflamation; • Cell type dependent biomodulatory activity	Drug repurposing • Novel activity profile of targeted therapies in the biomodulatory context; • Pleiotropic novel activities
• Targeting rationalizations of cancer hallmarks • Targeting modular events • Targeting the ‘metabolism’ of evolutionary processes • Targetring the holistic communicative context by implementation of non-normative boundary conditions	• Pioglitazoine (Actos) 45 mg p.o. daily • All-trans retenoic acid 45 mg^2^ p.o. daily • Interferon alpha 3MU s.c. three times a week Pioglitazone (Actos) 60 mg p.o. daily • Pioglitazone (Actos) 60 mg p.o. daily Dexamethasone 0.5 to 1.0 mg daily	• Treosulfan 250 mg twice daily • Trofosfamide 50 mg thrice daily • Capecitabine 1 g twice daily • 5-azacytidine 75 mg absolute 7 days, every 4 weeks	• Low-dose lenalidomide (after lenalidomide failure) 15 mg p.o. daily • Imatinib 400 mg p.o. daily • mTor inhibitor (in Hodgkin lymphoma, everolimus, serum level 15 ng/ml; in multivisceral Langerhans cell histiocytosis, temsirolimus weight-adapted in child) • COX-2 inhibitor etoricoxib 60 mg p.o. daily or rofecoxib 25 mg daily

### Dual Transcriptional Modulation

Ligands of nuclear receptors (NR) cue signaling response, establish ligand- nuclear receptor links to transcription- and signal transduction layers, induce gene expression changes, modulate cell type- and ligand-dependent cell fate transitions [17] and finally - so the present hypothesis—propose differential tumor- and involved organ response**.**

At the cell level, cell fate transitions comprise the regulation of a plethora of gene programs including, among others, regulation of cell proliferation, metabolism and specific functionalities that are acquired by differentiated cells. While the early steps of NR function and their impact on organ physiology is well understood, little is known about the dynamic gene networks that ultimately cause or modulate a particular pathophysiological phenomenon, e.g., a hallmark of cancer, supported by respective NR ligands/hormones in tumor tissues. Therapeutic dual transcriptional modulation aims at reprogramming communication tools in tumor tissues for attenuating tumor growth [11–13, 17].

### Communicative Reprogramming

To fill the gap in our understanding of cellular therapies in situ, particularly, on the activity profile of transcriptional modulators in complex tissue organizations, we operationalized the proposed communicative reprogramming of tumor tissues according to classifiable clinical phenomena arising during cellular therapies in situ.

Although, the systematization of tumor-associated communication tools is already a result of clinical observations during and after cellular therapies in situ, we introduce these tools up-front to present the respective clinical observations clearly arranged [15, 16].

The novel therapeutic tools for specifically designing communicative reprogramming (anakoinosis) focus on four topics [15], on redirecting (1) rationalizations of cancer hallmarks (the diverse physical constitutions of single cancer hallmarks) [18], (2) modular events (changing validity and denotation of systems objects) [19], (3) the ‘metabolism’ of evolutionary processes (the sum of extrinsically, i.e., therapeutically, and intrinsically inducible evolutionary processes within the tumor compartment) and (4) the holistic communicative context of structures, functions, decision maxims, i.e., hubs, in the tumor compartment, which finally determines validity and denotation of tumor promoting communication lines.

### Phase I/II Trials and Compassionate Use Therapies

Altogether, 144 patients (age ranging from 11 months to 83 years) suffering from six histologically different tumor types were treated in three published phase II trials, one phase I trial, and three compassionate use therapies (two already published) and are now available for studying response behavior to cellular therapy in situ: Two published phase II trials on metastatic renal clear cell carcinoma (RCCC), trial II (*n* = 31, 33 patients for intent-to-treat analysis) including pioglitazone and interferon-alpha; trial I (*n* = 18) including pioglitazone, serves as historic control [20]; two published trials on castration-resistant prostate cancer CRPC I (*n* = 38); CRPC II (*n* = 61) [21, 22], differing in the metronomically administered cytotoxic drug, capecitabine (CRPC I) versus treosulfan (CRPC II), and in an additional targeted therapy, imatinib (CRPC II); one published phase I part of an on-going phase II trial for multiple myeloma (MM, phase I, *n* = 6, > = third line therapy, retreatment with lenalidomide after IMiD failure was allowed) [23], and two already published retrospective evaluations (compassionate use) of patients with acute myelocytic leukemia (AML, *n* = 5) refractory to standard induction chemorefractory [24], or chemo- and brentuximab-vedotin-refractory classic Hodgkin lymphomas (cHL, *n* = 3, forth-line therapy) [25], and one, yet unpublished, pediatric compassionate use therapy for chemorefractory multivisceral Langerhans cell histiocytosis (third line therapy in mLCH, *n* = 2) in analogy to recently published data on mLCH [26, 27].

**The two consecutive eighteen and 11 months old children** (girl/boy) **with chemorefractory mLCH** were treated after informed consent by their parents on compassionate-use basis as indicated in Tables 1 and 2. Treatment was performed in the absence of alternative therapeutic options outside a clinical trial and without formal institutional review board approval.

**Table 2:** Cellular therapies in situ (trials and compassionate use programs) in pretreated or refractory hematologic malignancies or metastatic tumors

	Cellular therapy in situ						
Neoplasia	Therapy-line	No	Metronomic chemotherapy	Transcriptional modulator	COX-2 inhibitor	Small molecule	Publication
Renal clear cell carcinoma, Phase II (RCCC*I*)	Unlimited number of previous systemic therapies	18	*Capecitabine*	Actos	COX-2 inhibitor	–	*Biomarker Insights, 2006*
Renal clear cell carcinoma, Phase II, (RCCC*II*)	Unlimited number of previous systemic therapies	45	*Capecitabine*	Actos, IFNalpha	COX-2 inhibitor	–	*World J Urol, 2002*
Prostate cancer Phase II, (CRPC *II*) ClinicalTrilas.gov, NCT00427999	Castration-resistant, < 3 months PSA doubling-time in 77 %	61	Treosulfan	Actos, Dexa	COX-2 inhibitor	Imatinib	*Cancer Microenvironm. 2014*
*Prostate cancer,* Phase II (CRPC *I*)	Castration-resistant	36	*Capecitabine*	Actos, Dexa	COX-2 inhibitor	–	*World J Urol. 2013* *Lancet Oncology, 2006*
Langerhans cell histiocytosis, multivisceral (compassionate use)	Third-line, chemorefractory	2	Trofosfamide	Actos, Dexa	COX-2 inhibitor	−/+ Temsirolimus	*Br. J. Haematol, 2005*
Classic Hodgkin lymphoma (compassionate use)	Forth-line, chemorefractory	3	Treosulfan	Actos, Dexa	COX-2 inhibitor	Everolimus	*Br. J. Haematol, 2015*
Acute myelocytic leukemia (compassionate use)	Refractory to standard induction chemotherapy	5	*5-azacytidine*	Actos, Vesanoid	–	–	*Haematologica, 2014*
Multiple myeloma Phase *I*ClinicalTrials.gov, NCT001010243	> = 3rd-line, pretreatment with lenalidomide	6	Treosulfan	Actos, Dexa	–	Lenalidomide	*Blood 2012;* ***120*** *:5029*

**Some important activity profiles of anakoinosis inducing regimens could be only shown after withdrawal of patients from study** due to developing resistance (CRPC II trial, *n* = 10); or non-oncologic surgical interventions (knee or hip replacement in CRPC II trial, *n* = 6) in case of preceding prostate specific antigen response (PSA in serum <4 ng/mL). The surgical patients, all having rapidly progressive disease at study inclusion (PSA doubling time < 3 months), were followed without any tumor specific therapy, except bisphosphonates, until PSA doubling. Ten patients with CRPC were additionally treated with a gonadotropin-releasing hormone (GnRH) agonist, leuprorelinacetate, after developing resistance to cellular therapy in situ, despite the preceding resistance to GnRH agonists.

### Redirecting Rationalizations of Cancer Hallmarks

**Rationalizations** in the present context describe the multifold physical constitutions of single hallmarks of cancer [18]. The constitution of rationalizations comprises multiple and, if pathophysiologically required, exchangeable tumor cell compartments (redundancy) [28].

The following rationalizations for hallmarks of cancer were clinically monitored:

- **Inflammation response in serum:** In RCCC (RCCC trial I, II), mLCH and MM inflammation control was defined as a >30 % decrease of C-reactive protein from base-line [20, 23]. In cHL a normalization of CRP levels was considered critical as clinically relevant inflammation response [25].
- In CRPC we followed **immune response** in one patient with tumor-associated (paraneoplastic) lupus erythematosus (CRPC I) [29].

**The specific constitution of rationalization processes,** such as inflammation can be shown by inflammation control following specific dual transcriptional modulation (Tables 1 and 2):

- In the RCCC II trial pioglitazone was supplemented by low-dose interferon-alpha three times a week (Tables 1 and 2). Inclusion criteria in RCCC trial I, II were identical; therefore, a historic comparison of PFS and OS is possible [20].
- In a 2 years old boy with chemorefractory multivisceral Langerhans cell histiocytosis and insufficient inflammation control (fever) with pioglitazone / dexamethasone besides metronomic low-dose chemotherapy, **temsirolimus** was added in an adoptive therapy design in analogy to the therapy schedule for cHL.
- Heterogeneity of response to anakoinotic therapy: In the CRPC II trial PSA response was presented in waterfall plots to depict therapy sensitivity and resistance of single tumors [22].

### Rearranging Modular Structures

Communication technically, cells are artifacts with situate varying cellular identities and communication tools. **Modularity** - in a novel definition - describes the degree and specificity to which systems’ objects, i.e., cells, pathways, molecules, therapeutic targets etc. may be communicatively rededicated by **anakoinosis** that means, how they alter their validity and denotation in the systems context [15, 16].

Objective response of multiple pretreated, chemo- or castration-resistant tumor diseases to cellular therapy in situ may be achieved by **modular events**, that means without specifically targeting any tumor promoting pathway, but by communicatively rededicating validity and denotation of tumor-promoting systems objects.

- **Progression-free survival (PFS)** was the primary endpoint of all prospective trials and was also studied in the compassionate use projects.
- For CRPC I, II and RCCC I, II also **survival data** are available [20–22].
- **Tumor, leukemia and lymphoma cell death:**
  - **Histopathological evaluation of complete remission** (CR in bone, lung) could be performed in RCCC II [20].
  - **Molecular CR** (CRm) in AML: Leukemia cell death could be monitored by morphology and polymerase chain reaction (PCR) studies for molecular-genetic markers of AML blasts at diagnosis [24].
  - Histologic evaluation was possible at all metastatic sites in both children with mLCH.
  - Histologic and serologic confirmation of CR (serum and urine) in MM [23].
- **Disease chronification** at minimal residual disease (prostate-specific antigen, PSA < 4.0 ng/mL) was studied in CRPC II.
- **Delayed response** (partial remission, PR after > 4 months) in RCCC II and rapid response (<= 4 cycles, correspondingly 4 months) was monitored in chemorefractory cHL and AML [20, 24, 25].

### Therapeutically Mediated Diversification of Evolutionary Tools in the Tumor

The **specific ‘metabolism’ of evolutionary processes** in a tumor may be considered as the sum of intrinsically and therapeutically, via anakoinosis inducible evolutionary processes within the tumor compartment.

Intrinsically occurring tumor-associated evolutionary processes are characterized by the acquisition of molecular-genetic heterogeneity [30, 31]. Cellular therapy in situ claims to externally induce evolutionary processes via communicative reprogramming of cellular tumor compartments. Therefore, novel tumor systems behaviors may be clinically expected following induction of anakoinosis:

- **Induction of biological memory** means that tumor regrowth is significantly delayed in comparison to the corresponding rapid progression at study inclusion (PSA doubling time < 3 months), although study medication was withdrawn due to non-tumor related surgery. In CRPC II, PSA doubling time prior to study inclusion has been routinely studied.
- **Restoration of hormone-sensitivity** in CRPC was monitored following development of resistance to study medication by the add-on of leuprorelinacetate, while continuing study medication.
- Further, **sites of tumor response** in RCCC II, cHL, and mLCH were monitored. These observations give hints, whether therapy response can be established at **all** metastatic sites, despite the suggested molecular-genetic tumor heterogeneity at metastatic sites.
- At relapse or progression, **metastatic sites** were routinely monitored in the RCCC II trial to study, whether anakoinotic therapy approaches may attenuate metastatic spread [20].
- **Molecular-genetic heterogeneity and induction of anakoinosis:** In AML, response could be correlated with respective molecular-genetic aberrations at diagnosis [24].
- **Differentiation response:** In AML we studied granulocytes for molecular-genetic aberrations found in AML blasts at diagnosis [24].
- **Patients with CRPC** and lymph node metastases (CRPC II) were routinely screened for objective response with CT scans [22].

### Holistic Communicative Context in the Microenvironment Determining Specific Pathologies

Tumor diseases are specified by characteristic tumor histologies which arise on the basis of likewise tumor-specific communication tools, operating among tumor cells, their corresponding stromal surroundings and the respective organ site. Cellular therapies in situ, as top-down strategies interfere with those specific communication tools of a tumor, which are suggested to be holistically organized for constituting typical tumor-stroma-organ interactions.

**Holistic communicative conditions** require a communication-derived assessment of systems participators from the participator’s view: That means communication with a neighboring cell compartment in a tumor may not only alter validity and denotation of the addressee, but simultaneously of the addressor from the point of view of the addressee [15].

Therefore, **monitoring biologic aspects of tumor response** should uncover novel cytological or histological rearrangements or imaging technical behaviors (PET scan, bone scan) depicting reciprocal communicative interactions within tumor-stroma-organ compartments [22, 25].

**In case of AML** and **MM** bone marrow cytology and blood count provide insights in communicative interactions of leukemia cells and normal hematopoiesis [23, 24].

**In Hodgkin disease**, metabolic activity in the lesions **(PET** scan) could be correlated with CRP response in serum [25].

In the children with **mLCH** restoration of severely altered organ functions could be monitored, in **RCCC and CRPC** the CT-morphological behavior of osteolytic and osteoplastic metastases, respectively.

In CRPC II, technetium up-take in bone lesions was monitored **by technetium scans** during PSA response [22].

### Safety and Quality of Life

For all patients safety data are available and already published, except in case of mLCH (*n* = 2). Data are given in the present paper.

In CRPC data on quality of life during the first 6 months on cellular therapy in situ are available [22].

## Results

### Modulation of the Tool of Rationalizations Constituting Hallmarks of Cancer

#### Inflammation Control

**Fig. 1:**
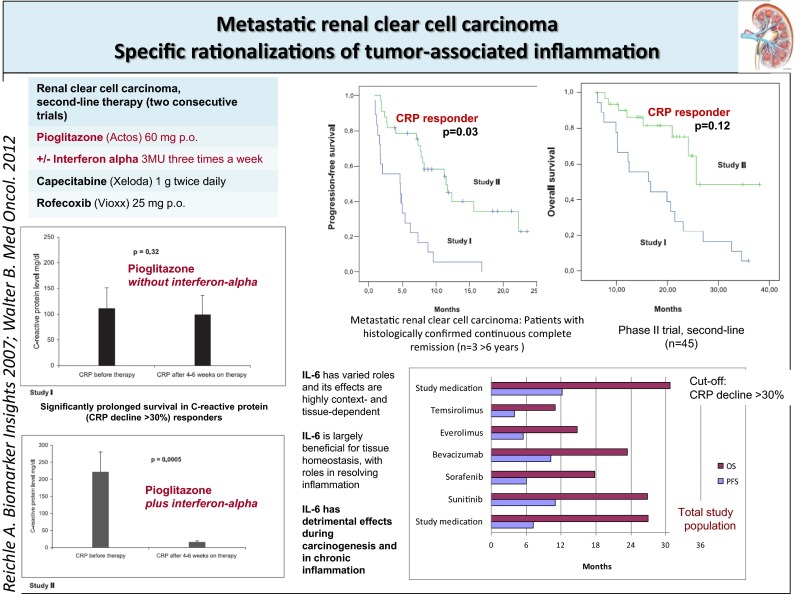
The addition of interferon-alpha to pioglitazone in pretreated renal clear cell carcinoma is associated with inflammation control, improved PFS and OS in comparison with a historical control. PFS and OS data compare with those achieved in first-line with standard therapies

**Fig. 2:**
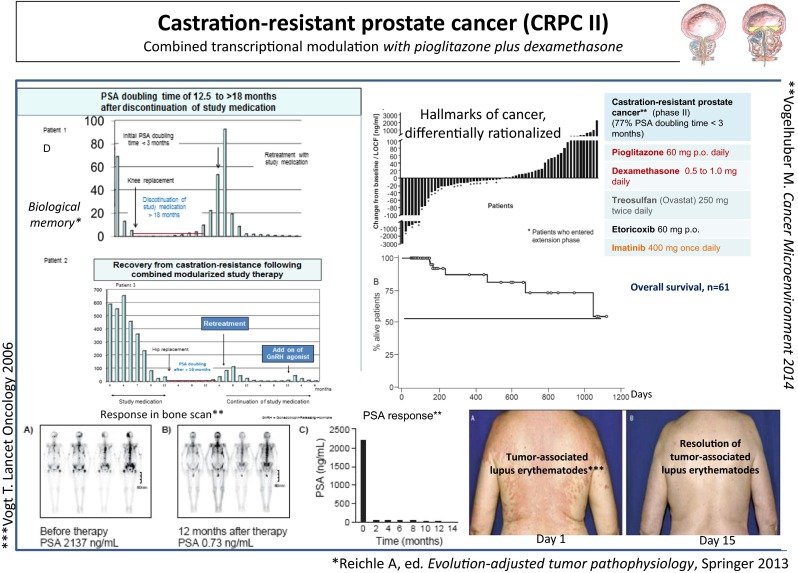
**A** In the CRPC II trial, median OS has not been achieved after 3 years, although 39 % of the patients did not show objective response (>50 % PSA response) to cellular therapy in situ. **B** Response in bone scan was observed in six of six patients. **C** Control of paraneoplastic lupus erythematosus was associated with objective tumor response. **D** Therapy response was on-going in six patients beyond stop of study medication (5 to 18 months) due to non-oncologic surgery. Following resistance to cellular therapy in situ, 60 % of the studied patients regained responsiveness against gonadotropin-releasing hormone (GnRH) agonists

A limited pattern of hallmarks of cancer could be clinically monitored**,** such as **inflammation**, via CRP response, in RCCC I/II (*n* = 18; *n* = 31) (Fig. 1), in MM (*n* = 6) and mLCH (*n* = 2), **immune response** in one patient with CRPC (CRPC I) (Fig. 2).

**Specification of anti-inflammatory therapy** in RCCC II by the add-on of low-dose interferon-alpha (Table 2) led to a significant decline of CRP levels in serum, to a significantly improved PFS and a tendency to improved OS in comparison to the respective historical control group, receiving pioglitazone alone (RCCC I) (Fig. 1) [22, 32–34].

**In MM** response (long-term stable disease, partial remission, very good partial remission and CR) was associated with >30 % CRP decrease (*n* = 6) [23].

**In cHL** the CRP levels rapidly normalized (*n* = 2) and PET negativity ensues at quite different metastatic sites, in lymph nodes, bone and lung lesions. One patient has shown no elevated serum CRP levels at study inclusion, but also achieved continuous complete remission cCR [25]. Long-term cCR was observed in two of three patients with cHL following allogeneic blood stem cell transplantation.

**Fig. 3:**
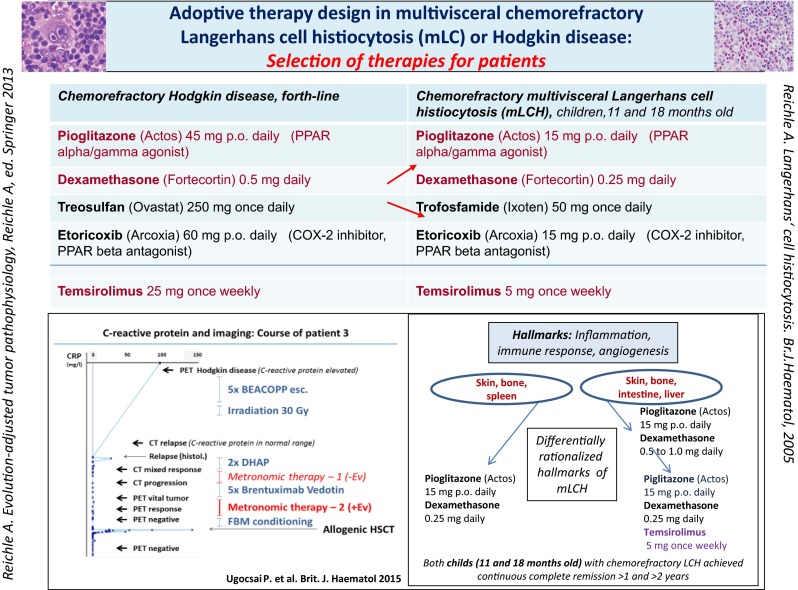
Left site showing C-reactive protein (CRP) follow-up during chemo- and brentuximab-vedotin refractory cHL. The add-on of everolimus to combined transcriptional modulation with pioglitazone and dexamethasone plus metronomic low-dose chemotherapy led to PET negativity and continuous CR following allogeneic blood stem cell transplantation. The right site indicates that chemorefractory mLCH may respond with cCR following cHL-therapy without everolimus. But patient two with chemorefractory mLCH needed the add-on of temsirolimus to achieve cCR

**In mLCH,** CRP levels normalized in response to compassionate use therapy (Fig. 3) [25].

#### Immune Response

Resolution of a tumor-accompanying systemic lupus erythematosus occurred in CRPC I (*n* = 1) within 14 days on treatment with study drugs. Immune response was paralleled with objective tumor response (Fig. 2) [29].

#### Communication Tools, Available for Induction of Anakoinosis may be Heterogeneous within a Histologically Defined Tumor Type

Waterfall plots for PSA response in the CRPC trial II (Fig. 2) indicate differential sensitivity of cellular therapy in situ: A percentage of 39 % CRPC patients did not respond to cellular therapy in situ (PSA decline in serum <50 %), nevertheless OS for the total patient population is nearly doubled compared to standard docetaxel therapy [22].

#### mLCH

In a **18 months old girl** with chemorefractory multivisceral LCH, weight-adapted metronomic low-dose chemotherapy plus pioglitazone and dexamethasone induced cCR (1 year+) in third-line (Fig. 3).

Only the **adoptive change** to pioglitazone/ dexamethasone **plus** temsirolimus, besides metronomic low-dose chemotherapy in third-line successfully resolved tumor-associated fever in a **11 months old boy** with chemorefractory mLCH, resulting in histologically proven cCR (2 years +). The successful therapeutic adoption indicates that inflammation/angiogenesis and immune response may be differentially rationalized within mLCH diseases [26].

### Therapeutically Exploiting Modular Events

The communicatively induced rededication of validity and denotation of tumor-associated systems participators led to objective responses in refractory (AML, cHL, mLCH, MM) or repetitively progressive malignant diseases, i.e., in patients with CRPC (77 % PSA doubling time < 3 months) [22], and patients with RCCC (RCCC II) [20].

**Long-term chronification of CRPC** with follow-up of >4 to 6 years on therapy is possible (*n* = 6), also in MM (>3 years VGPR, *n* = 1).

#### Velocity of Response

Objective responses occurred partly **very fast** (<= 8 weeks) in very rapidly progressive chemoresistant diseases, such as in CRPC (*n* = 10, PSA decline >50 %), AML (*n* = 5), cHL (*n* = 3), mLCH (*n* = 2), or in contrast, **very delayed** in RCCC II, after 4 to 6 months (10 of 15 responders, thereof *n* = 2 pathologic CRs).

**Tumor, leukemia and lymphoma cell death** (Table 3): In all refractory or multifold pretreated malignancies, cell death of malignant cells could be directly or indirectly monitored: In the trial **RCCC II,** cCR > 5 years (*n* = 4), long-term OS (>3 years, *n* = 7) and pathologically confirmed CR in bone (*n* = 2) [20], in **mLCH** histologically confirmed CR at all metastatic sites (*n* = 2, Fig. 3), CR in third-line of **MM** (1 of 6) [23], molecular CR (3 of 5 patients) and cCR (*n* = 1) in **AML** [24], and in **Hodgkin lymphoma** PET negative PR (*n* = 3) [25]. In two of 13 patients with lymph node involvement, treated in **CRPC II trial,** calcifications in involved lymph nodes were found in CT scans as indicators for tumor necrosis [22].

**Table 3:** Indicating best responses to cellular therapies in situ

Anakoinosis inducing therapy elements: Dual transcriptional modulation and best response			
Transcriptional modulators	Metastatic diseases	Remission induction? Best response	Add-on of transcriptional modulators/repurposed targeted therapies
Pioglitazone plus dexamethasone	• *Castration-resistant prostate cancer* • Multiple myeloma *(third-line)* • Multivisceral Langerhans cell histiocytosis *(chemo-resistant)* • Hodgkin lymphoma *(forth-line)*	*Minimal residual disease 6 years+* First CR in third-line, VGPR 33 months+ Continuous complete remission (histologically Confirmed CR) PET negativity	• Response after lenalidomide failure • Everolimus • **Temsirolimus**
Pioglitazone plus interferon-alpha	• Renal clear cell carcinoma *(second-line)*	Continuous complete remission (Histologically confirmed CR)	• **Interferon-alpha**
Pioglitazone plus all-trans retinoic acid	• Acute myelocytic leukemia *(refractory)*	Continuous complete remission (molecular CR)	• After 5-azacytidine failure rapid response

**In CRPC II,** OS did not achieve the median at 36 months despite the negative selection of patients with 77 % PSA doubling time < 3 months before study inclusion. The median OS for standard therapy with docetaxel is 19 months [35].

#### Survival in Pretreated Patients with RCCC (RCCC II) Compares with Historic Controls Treated in First-Line with Standard Therapies

Median overall survival at 3 years was 5 % in RCCC trial I versus 48 % in RCCC II, which compares with first-line OS rates to standard therapies (Fig. 1) [20].

### Targeting the Specific Metabolism of Evolutionary Processes

Cellular therapy in situ enables induction of qualitatively novel tumor systems’ behaviors, as exemplarily shown by induction of **differentiation** in non-acute promyelocytic leukemia (**non-APL)** patients (*n* = 2) [24]; of **biological memory** (CRPC II, *n* = 6), and **reconstitution of hormone responsiveness** in CRPC (six of ten patients), and **reduction of the metastatic potential** in RCCC II (21 of 31 patients, 68 % progression at the original metastatic sites) [16].

- **Biological memory:** Long-term chronification of CRPC (CRPC II) after withdrawal of study medication (*n* = 6) reveals that dual transcriptional modulation may operate evolutionary processes by simultaneously establishing robustness and homeostasis at the novel evolutionary level (biological memory) (Fig. 2). It took 5 to 18 months until PSA doubling. Transcriptionally induced reprogramming of tumor compartments on an epigenetic level seems to play a major role for inducing biological memory.
- **Restoration of hormone sensitivity:** Six out of ten patients with progressive disease on study medication have shown a more than 50 % PSA response after addition of a gonadotropin-releasing hormone (GnRH) agonist and continuation of study medication.
- **Differentiation:** A novel phenomenon in AML was a long-lasting ‘defect healing’ (>6 and 9 months disease stability), characterized by maturation of **non-APL blasts** in granulocytes with corresponding molecular-genetic aberrations of the blasts (*n* = 2) [24].

#### Genetic Heterogeneity and Anakoinosis

Observations in RCCC II, AML, and cHL, together with the successful adoptive therapy design in mLCH, give hints that

**Fig. 4:**
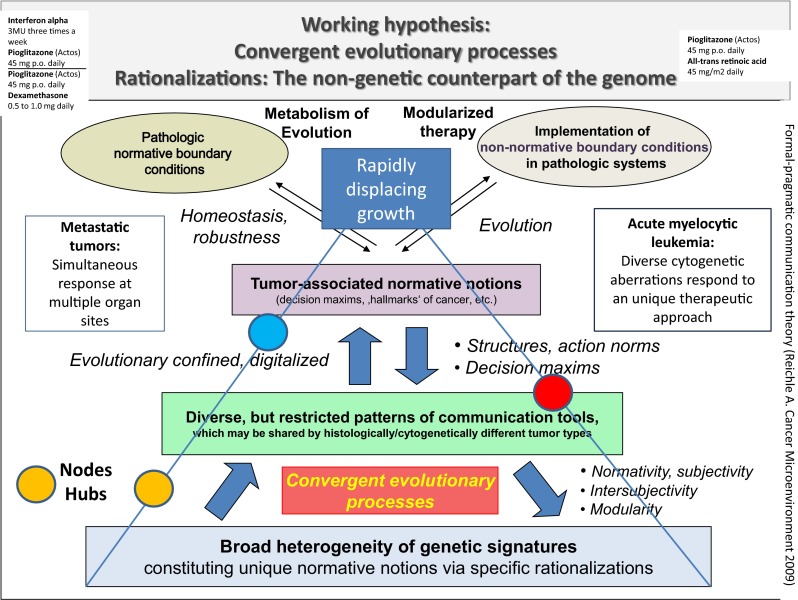
Simultaneous tumor response at multiple metastatic tumor sites and response of cytogenetically heterogeneous AMLs to a unique therapeutic approach indicates that restricted pattern of communication tools, particularly rationalizations of hallmarks of cancer, are uniquely accessible with cellular therapies in situ

- Cellular therapy in situ may successfully address molecular-genetic heterogeneity at metastatic sites, which is commonly associated with mixed tumor response to classic targeted therapies, by concertedly designing still preserved and uniquely constituted communication tools at metastatic sites: Mixed responses in metastatic RCCC II occurred rarely (23 %). No mixed responses were observed in cHL and mLCH despite the suggested genetic heterogeneity at metastatic sites.
- Despite molecular-genetic and genetic heterogeneity in AML, these patients responded—even though qualitatively different - to a unique therapeutic approach, at best with molecular remission. This observation indicates the presence of convergent evolutionary processes accessible to anakoinosis (Fig. 4) [24].

#### Targeting the Holistic Communicative Context in the Microenvironment

**Fig. 5:**
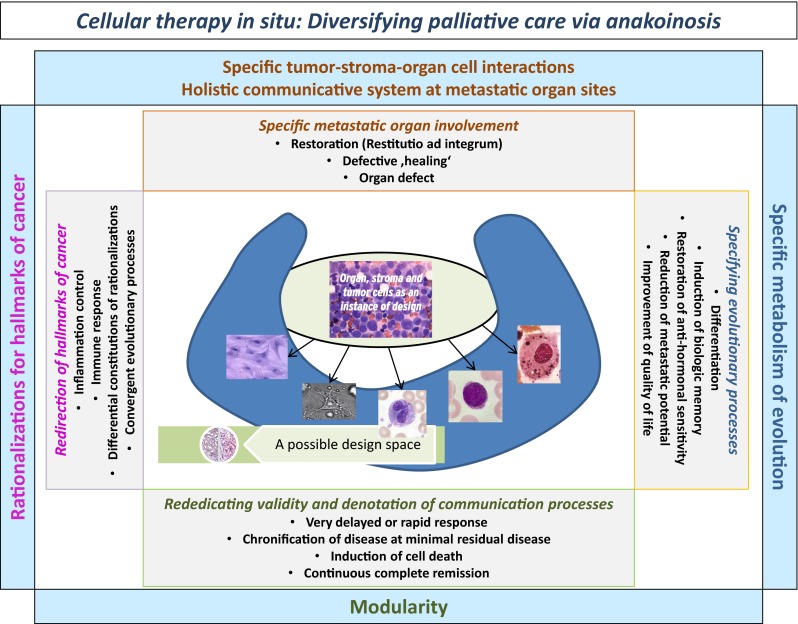
Cellular therapies in situ get access to the tumor’s design space via anakoinosis

Due to the holistic communicative context in the microenvironment, ‚seed and soil’ are reciprocally exchangeable as indicated by the implementation of novel communicative reprogramming inducing boundary conditions, i.e., cellular therapy in situ (Fig. 5, Tables 4 and 5). Induction of response via non-theme-dependent targets reveals that **the communicative context** of reductionist derived structures, functions, and decision maxims, i.e., regulatory acting hubs, are equally important in determining validity and denotation of tumor promoting communication lines as reductionist attributed functions of tumor systems objects.

**Table 4:**
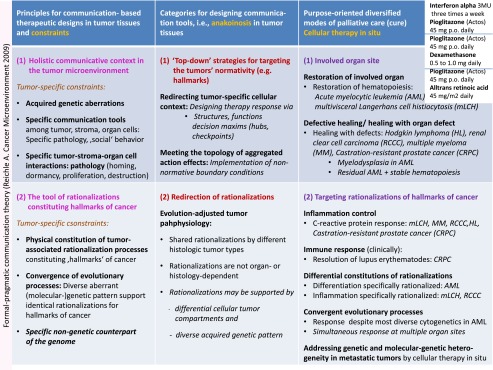
The left column presents the two of four communication tools (the holistic communicative context in the tumor microenvironment andthe tool of rationalizations  constituting hallmarks of cancer) and  their corresponding constraints, which specify the respective communication processes. The middle column shows the communication technical targets operated by anakoinosis. The right column summarizes therapeutic tools accessible by anakoinosis

**Table 5:**
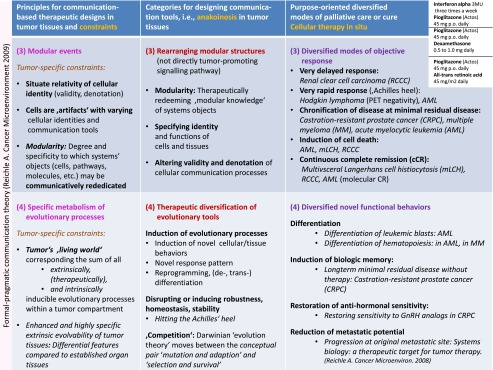
The left column presents the two of four communication tools (modular events and the specific metabolism of evolutionary processes) and their corresponding constraints, which specify the respective communication processes. The middle column shows the communication technical targets operated by anakoinosis. The right column summarizes therapeutic tools accessible by anakoinosis

As a consequence, cellular therapies in situ induce specific functional changes likewise in tumor, adjacent stroma and organ cells:

- Rapid restoration of the involved organ site, e.g., bone marrow, could be achieved in AML with induction failure, in 3 of 5 patients. With the administration of low-dose 5-azacytidine, neutrophil counts in the peripheral blood increased at each start of a 4 weeks cycle [24]. In contrast, the approved cytotoxic doses of azacytidine are associated with a decline of neutrophils in the peripheral blood [36].
- Unchanged, osteolytic ‘defect healing’ could be observed in RCCC and MM. In CRPC osteoplastic metastases remained stable, also in case of PSA response.
- In mLCH skin lesions (*n* = 2) and severe mLCH associated bone marrow insufficiency (*n* = 2) resolved, however, following liver failure due to infiltration, the liver function improved with severe defects (*n* = 1), but lesions in the intestine completely resolved (*n* = 1). The boy was originally designated for combined liver and bone marrow transplantation.
- Negative PET scan revealed significant metabolic changes in cHL (*n* = 3) during therapy despite the presence of residual disease in CT scans [25].
- Missing technetium up-take despite the unaltered presence of osteoplastic metastases in CT scan was detected in 6 of 6 PSA responders (CRPC II) [22].
- In MM, hemoglobin levels increased in 4 of 6 pts > 1.0 g/dl during the 4 weeks core phase. That means hemoglobin increase could not be due to rapid myeloma clearance in the bone marrow [23].

### Dual Transcriptional Regulation Stimulates Globally, Acts Locally: Toxicity Profiles of Schedules Including Dual Transcriptional Modulation are Modest

In **CRPC** toxicities grade 3 to 4 compare to abiraterone, but no cardiotoxicity occurred. Quality of life measurements during the first 6 months on therapy showed a stabilization of all functions and no impairment compared to pulsed chemotherapy (docetaxel) [22].

In **RCCC trial I, II** reported maximal toxicity was WHO grade 3, predominantly due to hand-foot-syndrome (capecitabine) [20].

- AML: Compared with conventional myelosuppressive chemotherapy regimens, treatment with 5-azacytidine, pioglitazone and all-trans retinoic acid was associated with much lower toxicity and could be easily administered in an outpatient setting. The most common adverse events were hematologic toxicities grade 1–4 without the need for platelets or erythrocyte transfusions or administration of growth factors [24].
- CHL: Compared with conventional myelosuppressive chemotherapy regimens, or brentuximab-vedotin therapy, the treatment was associated with much lower toxicity and could be easily administered in an out-patient setting without any hospitalization during therapy. Even no grade 3 or 4 hematologic toxicity occurred and there was no need for platelets or erythrocyte transfusions or administration of growth factors despite the heavily pretreatment of the three patients. Also no grade 3 or 4 non-hematologic toxicities were observed. Dose reductions were not necessary [25].
- MLCH: In the 11 months old boy high fever rapidly resolved after start of cellular therapy in situ including temsirolimus. Maximal toxicity observed was grade 3 hematotoxicity. Due to grade 3 hematotoxicity daily weight adapted trofosfamide doses were reduced in both patients.
- MM: Patients experienced no dose-limiting toxicity at both dose levels of lenalidomide (10 or 15 mg daily). All patients continued treatment in the extension phase. Serious adverse reactions observed during follow-up were infections (*n* = 6, Grade 2 NCI-CTCAE toxicity), depressed mood (*n* = 1, Grade 2), osteonecrosis of the jaw and tooth extraction (*n* = 1), and thrombosis during anticoagulation (*n* = 1, Grade 2) [23].

### Cellular Therapies In Situ

#### Dual Transcriptional Modulation

Pioglitazone was used in combination with dexamethasone or all-trans retinoic acid or interferon-alpha.

- In AML the add-on of pioglitazone to all-trans retinoic acid led to a novel behavior of leukemia cells (differentiation, leukemia cell death) and normal hematopoiesis (release of neutrophils in peripheral blood) [24]. In RCCC (RCCC II), the add-on of interferon-alpha decisively improved outcome, based on inflammation control [20].
- **Induction of anakoinosis** with identical pairs of transcriptional modulators, pioglitazone and dexamethasone (Tables 1 and 2), mediated multifaceted clinical responses in CRPC (CRPC II), cHL, MM and mLCH (Tables 4 and 5). Responses turned out to be heterogeneous in CRPC (CRPC II) (waterfall blot, Fig. 2) [22].

Metronomic low-dose chemotherapy:

- Changing the drug for metronomic low-dose chemotherapy, from capecitabine to treosulfan, improved in a historic comparison both, median PFS and OS from 4.0 to 14.1 months, respectively, in the CRPC I trial, to a PFS of 15.3 months and median OS, which has not been achieved after 3 years (CRPC II trial) [21, 22].

Drug repurposing:

- Imatinib did not add any additional clinical benefit, even in case of scheduled dose escalation (double dose) [22].
- However, either in cHL or in mLCH the add-on of an mTor inhibitor could induce PET negativity and PR (cHL) or continuous, histologically confirmed CR in mLCH [25].
- Continuing a low-dose IMiD therapy within a cellular therapy in situ after failure of IMiDs, may rescue MM patients (*n* = 1) [23].
- The impact of COX-2 inhibitors could not be evaluated in the present designs of cellular therapies in situ.

Adoptive therapy

- **For adopting anakoinotic constellations**, drugs with anakoinotic activity profile were selected (drug repurposing); in AML low-dose azacytidine (epigenetic reprogramming) [24], in mLCH or cHL, mTOR inhibitors (immune modulatory, angiostatic) [25], in MM, lenalidomide (immune modulatory) [23], and in several tumor types COX-2 inhibitors (anti-inflammatory) (Table 2). Also the chosen cytotoxic drugs for metronomic chemotherapy seem to impact outcome (CRPC I/II).
- **Adoptive therapy** in an individual patient was successfully used in mLCH and cHL (Fig. 3), in a patient cohort in RCCC (RCCC II).

## Discussion

### Diversified Modes of Palliative Care or Induction of Cure by Cellular Therapy In Situ

Distant metastasis is the leading cause of cancer mortality. Context-dependently changing validity and denotation of tumor-promoting pathways [19, 30, 31, 37, 38], and genetic heterogeneity at metastatic sites are major reasons for failure of theme-dependent (pathway-,epitope-directed) targeted therapy [39–41].

Communicative reprogramming, i.e., anakoinosis, induced by cellular therapies in situ turned out to consistently overcome classic intrinsic, extrinsic resistance, and particularly resistance due to (molecular-) genetic tumor heterogeneity, as indicated by multiple modes of long-term tumor control, even in small series of consecutively treated patients suffering from refractory, metastatic tumor diseases or hematologic neoplasia with quite different histologic origin. Cellular therapies in situ successfully overcome resistance by concertedly redirecting **convergent, but differentially organized communication tools** (Fig. 4), while been supported by quite different pattern of chromosomal and molecular-genetic aberrations [24, 40, 41].

Induction of anakoinosis in chemorefractory metastatic neoplasia is characterized by diversified response patterns along the trajectory of tumor-specific communication tools. The conceptual introduction and operationalization of communication tools, which allows redirecting validity and denotation of tumor-promoting systems participators (cells, pathways etc.), is the starting point for the therapeutic turnaround from a classic theme-dependent to a communication oriented therapy strategy (Tables 4 and 5) [19].

### Anakoinosis in Tumor Tissues: Categories for Designing Communication Tools

For the first time, to our knowledge, comprehensive categories for designing anakoinosis could be separated (Tables 4 and 5) on the basis of systematic clinical observations during cellular therapies in situ and after withdrawal due to tumor progression or non-oncologic surgical interventions. The summarized principles for communication- based therapeutic designs close the gap, between available results describing communication design on a molecular level in simplistic systems in vitro, for example with transcriptional modulators [11–13, 17], and the clinical observations derived on the scale of complex tissue levels in refractory tumors**.**

**Anakoinosis** describes the therapeutic accessibility of reciprocal communicative interactions among biologic systems participators, i.e., structures (cell compartments, pathways etc.), functions (angiogenesis, immune response etc.) and decision maxims (cellular hubs) via ubiquitously available, and as shown, specifically targetable communication tools (Tables 4 and 5, Fig. 7). The presented multifold therapeutically designed anakoinotic systems stages allow reconsidering what tumor systems have in common, namely targetable, seemingly elusive communication tools. Similar to immune modulating therapies [42], the anakoinotically induced systems stages would not spontaneously develop via natural evolutionary events.

An important observation is that normal organ systems are obviously not as susceptible to anakoinosis as tumor systems (Fig. 5). This is indicated by modest side effects of cellular therapies in situ, even during long-term administration (CRPC II, MM). Vice versa, tumors are downright characterized by anakoinotic accessibility. **Anakoinotic response profiles** can be now clinically categorized for therapeutically designing communication tools (Tables 4 and 5) [15].

**Tumor-associated rationalizations** constituting hallmarks of cancer are part of targetable communication tools [18]. Particularly rationalizations for immune response are already systematized: Identical tumor immune responses may be found at primary and metastatic sites [43, 44], and the kind of immune response is predictive for survival [45].

Classic targeted therapies single out specific rationalizations as important targets for tumor control [46, 47]. All these therapies leave out of consideration that rationalizations of hallmarks of cancer may have large variations in their constitution, and are often redundantly organized, such as angiogenesis [7], inflammation [16, 28, 48], and immune response [44–46, 49] etc.

Just specific anakoinosis inducing schedules meet these variations among the tumor-specific tools of rationalizations by adoptive therapy approaches: This tool represents the targetable, communication based non-genetic counterpart of the genome (Tables 4 and 5).

The architectures of communication tools (Tables 4 and 5) or rationalizations for hallmarks of cancer have in common that they may be unrelated to histologic tumor types or clinical stages [44, 45], and - therapeutically decisive—are often similarly organized at primary tumor sites and metastatic lesions (rare mixed responses in RCCC II [16, 44].

**Fig. 6:**
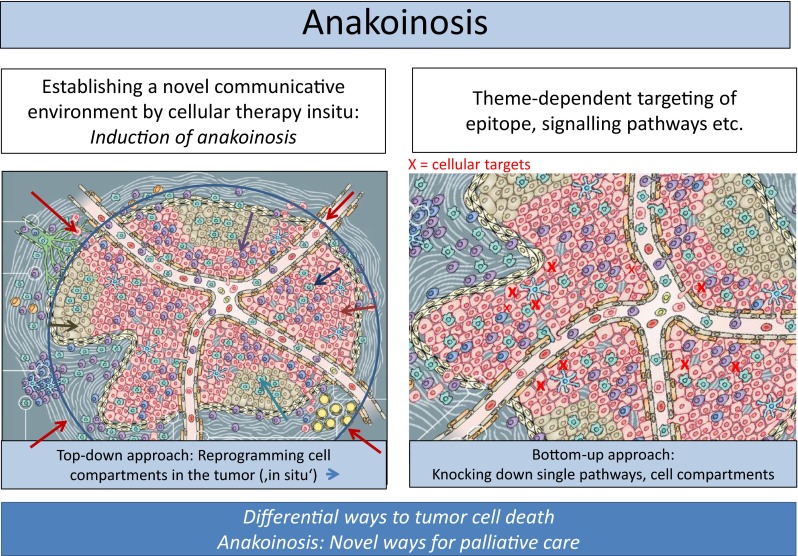
Bottom-up and top-down strategies both redirect and modulate rationalizations constituting hallmarks of cancer, but are using separate techniques, either theme-dependent targets or communicative reprogramming, i.e., anakoinosis

Common anakoinotic processes among tumors, unique constitutions of hallmarks of cancer in primary tumors and metastatic sites as well as shared anakoinotic processes within different histologic tumor types [7, 44, 45] support the claim for an **evolution-adjusted tumor pathophysiology** for guiding anakoinotic and rationalization oriented tumor therapies (Fig. 6) [50].

### Designing Cellular Therapies In Situ

The therapeutic elements of cellular therapies in situ are establishing ‘on-site’ novel boundary conditions, thereby, accomplishing a virtual communicative context for reprogramming tumor systems—importantly, in a reproducible and clinically relevant way (Tables 4 and 5).

**Fig. 7:**
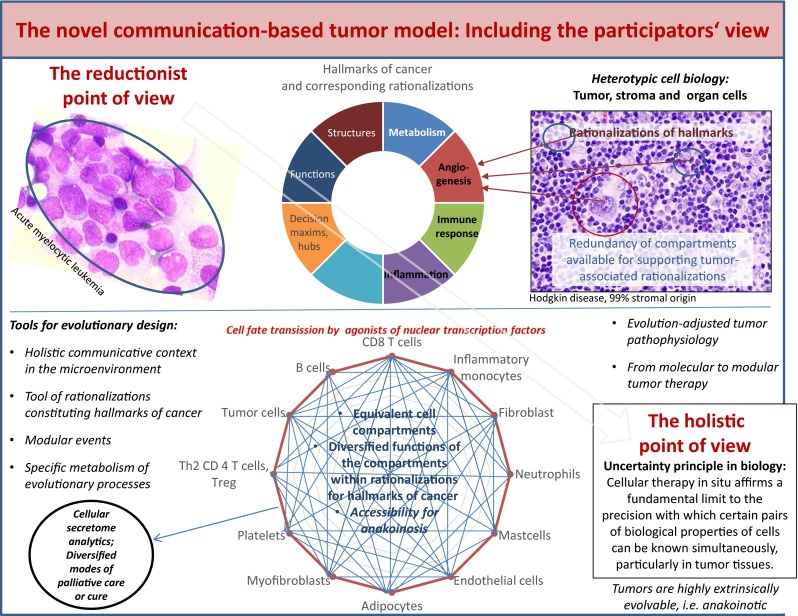
Anakoinosis inducing regimen may rescue refractory malignancies regardless of whether tumors are predominantly consisting of non-tumor cells, like in cHL, or of tumor cells, like in case of AML. Traditionally, reductionist tumor models are focusing separately on tumor and/or stroma cells (‘seed and soil’). Holistic models are aiming at the communicative system, equivalently constituted by tumor and stroma cells. The heterogeneous activity profiles of anakoinosis, even within a single tumor disease, now figures out fundamental limits to the precision with which certain pairs of biological properties in the cell compartments can be predicted simultaneously, particularly in tumor tissues. Comprehending and monitoring cell identities and functions during various systems stages of tumors will be of major interest in future for establishing anakoinosis and should stimulate intensified research in the field of cellular secretome analytics in blood serum or plasma

**Dual transcriptional modulation** emerged as pivotal therapeutic instrument for reprogramming tumor systems and for uncovering and therapeutically exploiting basic communication tools (Tables 1 and 2). Therapies designing communication tools equivalently target stromal and tumor cells, which are now considered as holistic communicative system (Fig. 7). Specific anakoinotic processes may be induced - as shown - regardless of whether tumors are predominantly consisting of stromal cells, like in cHL, or of tumor cells, like in case of AML (Fig. 7).

#### Drug Repurposing

The paradigmatic shift from low-dose metronomic dosing and considering cancer as targetable communicative system will be given priority in our approach to the selection of drugs for repurposing. The intention is not to look for new anti-proliferative cytotoxic agents or classic targeted therapies that can be used at maximum tolerated doses, the ‘magic bullets’, but to look for drugs from the available clinical tool that can be used to support the redirection of tumor-specific communication tools. There is a clear rationale for introducing combinations of regulatory acting agents, which concertedly work on multiple communication tools as indicated in Tables 4 and 5. The concerted regulatory activity may even evolve tumor systems for generating novel systems behaviors (Fig. 5).

**Many targeted therapies**, also those used in the current trials, may be repurposed, as their activity profile is strongly dependent on the tumor type and stroma composition, and - as shown by cellular therapies in situ - on the co-regulatory activity profile of the combination partners [9, 51]. In anakoinotic therapy schedules, the sum of single known drug activities may establish a completely novel quality of action in the tumor.

**Metronomic low-dose chemotherapy** is much better understood from its diverse interactions with rationalizations of hallmarks of cancer, such as immune response and angiogenesis [14] and may support the activity profile of transcriptional modulators [16, 52, 53]. Also the activity profile of low-dose metronomic chemotherapy is strongly dependent on the histologic tumor type or the tumor-specific communication tool, respectively [48].

**5-azacytidine** at low doses induces differentiation and activation of silenced genes. The clinical effects in non-APL AML patients treated with 5-azacytidine, all-trans retinoic acid and pioglitazone do not emerge in patient treated with the single drugs or as add-on to chemotherapy [54, 55]: Even 5-azacytidine at standard dose may be rescued by the triple combination, and patients refractory to standard induction therapy responded with CRm [24].

### Principles for Communication- Based Therapeutic Designs in Tumor Tissues and Constraints

Anakoinotic ‘operations’ may be summarized as **principles for communication- based therapeutic designs in tumor tissues and their corresponding tumor-specific constraints** (Tables 4 and 5)**.** Up-to-now the kind of anakoinotic reactivity can be only poorly predicted on the basis of molecular parameters. Therefore, we defined principles for communication- based therapeutic designs by defining constraints for design. The constraints are characterized by communication specifying key parameters as indicated in Tables 4 and 5.

In contrast to the commonly used reductionist definitions for modularity (the degree to which collective functions of systems participators are insulated from other components and pathways [56]), we use now an exclusive **communication-technical definition for modularity** (communicative rededication of validity and denotation of systems participators [15]).

On the background of the novel definition and the systematization of communication principles in tumors, therapeutic communication design represents a novel pathophysiologically relevant tool, even for inducing tumor cell death in resistant disease, besides multifold beneficial palliative treatment effects (Tables 4 and 5).

**Therapeutically specifiable anakoinotic constellations** in metastatic tumors may be integrated in novel systems-biological models to develop strategies for modifying tumor-associated communication tools with **adoptive cellular therapies in situ** (Fig. 7).

Therapeutically important is the fact that anakoinotic communication tools may be shared by histologically unrelated tumors [7] and by metastatic sites within an individual tumor disease [16, 44]: Anakoinotic response to dexamethasone plus pioglitazone was established in cHL, LCH, CRPC, and MM, and to pioglitazone plus all-trans retinoic acid in AML and pre-clinically in breast cancer [11, 24]**.** However, the **phenotypic expression of anakoinosis** among different histologic tumor types is qualitatively different as indicated by characteristic clinical features: The communicative background in the respective tumor specifically determines the phenotypic facets of anakoinosis.

The molecular basis of processes inducing anakoinosis needs to be evaluated further, particularly anakoinotic constellations leading to tumor cell death.

### Uncovering Tumor Systems Biology by Anakoinosis

In future, novel tumor models (Fig. 7) will define communication-technically exploitable resources for promoting anakoinosis with cellular therapies in situ: Top-down - in contrast to bottom-up approaches (Fig. 6) - reconsider communicative prerequisites determining situate validity and denotation of single tumor-promoting pathways or targets, the contingently organized communication tools (Fig. 4), and the **uncertainty principle** concerning the situate function of cellular tumor compartments (Fig. 7)—as shown for example in the diversified anakoinotic responses in AML, mLCH or in the CRPC II trial.

The heterogeneous activity profiles of anakoinosis inducing regimen, even within a single histologically defined tumor disease, now figure out fundamental limits to the precision with which certain pairs of biological properties in cell compartments can be predicted simultaneously, particularly in tumor tissues and different tumor sites. Tumor-associated communicative constellations provided for anakoinosis, should be consecutively analyzed, analytically or empirically at the bench and may be retranslated into new communicative systems interpretations. Thus, the methodology may partially reverse the traditional information flow, which is affected by the predominant transfer from analytical sciences to applied sciences [57].

Comprehending and monitoring cell identities and functions during various systems stages of tumors will be of major interest in future for establishing purposefully anakoinosis and should stimulate intensified research in the field of cellular secretome analytics in blood serum or plasma [8, 58].

Differential selection of specific anakoinotic processes for reprogramming tumor cell compartments may offer completely novel possibilities for diversifying therapeutic instruments aiming at long-term tumor control. These promising data with anakoinosis inducing combination therapies in chemo-refractory neoplasia warrant further investigation in clinical trials. Perspectival, also patients at risk for acquiring tumor diseases could benefit from novel anakoinosis inducing schedules.
